# A functional genetic screen defines the AKT-induced senescence signaling network

**DOI:** 10.1038/s41418-019-0384-8

**Published:** 2019-07-08

**Authors:** Keefe T. Chan, Shaun Blake, Haoran Zhu, Jian Kang, Anna S. Trigos, Piyush B. Madhamshettiwar, Jeannine Diesch, Lassi Paavolainen, Peter Horvath, Ross D. Hannan, Amee J. George, Elaine Sanij, Katherine M. Hannan, Kaylene J. Simpson, Richard B. Pearson

**Affiliations:** 1 Division of Cancer Research, Peter MacCallum Cancer Centre, Melbourne, VIC Australia; 2 Sir Peter MacCallum Department of Oncology, University of Melbourne, Melbourne, VIC Australia; 3 Victorian Centre for Functional Genomics, Peter MacCallum Cancer Centre, Melbourne, VIC Australia; 4 Josep Carreras Leukaemia Research Institute, Badalona, Barcelona Spain; 5 Institute for Molecular Medicine Finland (FIMM), HiLIFE, University of Helsinki, Helsinki, Finland; 6 Synthetic and Systems Biology Unit, Hungarian Academy of Sciences, Szeged, Hungary; 7 John Curtin School of Medical Research, Australian National University, Canberra, ACT Australia; 8 School of Biomedical Sciences, University of Queensland, Brisbane, QLD Australia; 9 Department of Biochemistry and Molecular Biology, University of Melbourne, Melbourne, VIC Australia; 10 Department of Biochemistry and Molecular Biology, Monash University, Clayton, VIC Australia; 11 Department of Clinical Pathology, University of Melbourne, Melbourne, VIC Australia

**Keywords:** Oncogenes, Tumour-suppressor proteins

## Abstract

Exquisite regulation of PI3K/AKT/mTORC1 signaling is essential for homeostatic control of cell growth, proliferation, and survival. Aberrant activation of this signaling network is an early driver of many sporadic human cancers. Paradoxically, sustained hyperactivation of the PI3K/AKT/mTORC1 pathway in nontransformed cells results in cellular senescence, which is a tumor-suppressive mechanism that must be overcome to promote malignant transformation. While oncogene-induced senescence (OIS) driven by excessive RAS/ERK signaling has been well studied, little is known about the mechanisms underpinning the AKT-induced senescence (AIS) response. Here, we utilize a combination of transcriptome and metabolic profiling to identify key signatures required to maintain AIS. We also employ a whole protein-coding genome RNAi screen for AIS escape, validating a subset of novel mediators and demonstrating their preferential specificity for AIS as compared with OIS. As proof of concept of the potential to exploit the AIS network, we show that neurofibromin 1 (NF1) is upregulated during AIS and its ability to suppress RAS/ERK signaling facilitates AIS maintenance. Furthermore, depletion of NF1 enhances transformation of p53-mutant epithelial cells expressing activated AKT, while its overexpression blocks transformation by inducing a senescent-like phenotype. Together, our findings reveal novel mechanistic insights into the control of AIS and identify putative senescence regulators that can potentially be targeted, with implications for new therapeutic options to treat PI3K/AKT/mTORC1-driven cancers.

## Introduction

The PI3K/AKT/mTORC1 pathway integrates signals from growth factor stimulation, nutrient and energy status, hypoxia and cellular stress to control cell growth, proliferation and survival, cellular metabolism, migration, and angiogenesis [1]. Recurrent PI3K/AKT/mTORC1 alterations, particularly activating hotspot *PIK3CA* mutations (E545K and H1047R) or inactivating *PTEN* mutations, occur in 30% of human sporadic tumors [2, 3]. Some (1.4–8%) breast, colorectal, and ovarian cancers display an *AKT*-E17K mutation conferring constitutive activation [4]. Furthermore, pathway hyperactivation occurs in breast (20–55%), lung (~60%), prostate (50%), and melanomas (40–70%) [5], driving preneoplastic lesions in lung, breast, and endometrial cancers [6–8]. Nevertheless, malignant disease progression requires additional genomic hits. Understanding deregulated signaling in normal cells will unveil key regulatory mechanisms that, if subverted, would allow malignant progression and identify therapeutic opportunities if these tumor-suppressive “brakes” were restored.

Excessive RAS/ERK pathway activation driving oncogene-induced senescence (OIS) is one such brake [9–12]. OIS is well characterized, featuring p16 upregulation, a persistent DNA damage response (DDR), senescence-associated heterochromatic foci (SAHF), enhanced SA-beta-galactosidase (SA-ßGal) activity, a SA-secretory phenotype (SASP), and cell cycle arrest [9, 13, 14]. However, additional genetic and epigenetic perturbations can reverse OIS to promote cancer development [15, 16]. Lymphocytes in Eµ-*Nras* mice display strong SA-ßGal activity; however, loss of the histone methyltransferase *Suv39h1* impairs SA-ßGal activity and causes T-cell lymphoma [10]. Constitutively active *Braf*^*V600E*^ promotes senescence in melanocytic nevi, but *Pten* depletion drives melanomagenesis [17]. Investigating OIS has also uncovered targeted therapy resistance mechanisms. Neurofibromin 1 (NF1) deficiency overcame *Braf*^*V600E*^-induced senescence and conferred BRAF inhibitor resistance in melanoma [18, 19].

We and others have demonstrated chronic PI3K/AKT/mTORC1 pathway activation promotes an OIS-like phenotype, termed AKT-induced senescence (AIS) [20–22], with PIK3CA mutants, PTEN knockdown, and constitutively active AKT triggering AIS [20]. Unlike OIS, AIS lacked a hyperproliferative burst and DNA damage, and proliferative arrest relied on p53 rather than p16. Increased p53 expression depended on mTORC1-dependent mRNA translation and MDM2’s nucleolar sequestration. Similarly, activated *Akt* or *Pten* knockout in murine prostate epithelium promoted intraepithelial neoplasia and senescence marker expression [21, 23], while subsequent *Cdkn1b* or *Trp53* loss facilitated adenocarcinoma progression and senescence marker loss. We hypothesize that, like OIS, AIS is a reversible tumor-suppressive mechanism, and understanding how it is overcome will identify mechanisms of tumorigenesis and therapeutic resistance in the 38% of human solid cancers with deregulated PI3K/AKT/mTORC1 activity [3].

Here, we provide insight into AIS using transcriptomic and metabolomic profiling, demonstrating overlapping signatures with OIS. We reveal suppression of RAS/ERK signaling is a unique AIS hallmark. Moreover, we screened the protein-coding genome and identified numerous escape routes beyond p53 loss. Employing genetic approaches, we validated high-confidence candidates in common with OIS and specific for AIS, demonstrating NF1-mediated suppression of RAS-ERK signaling maintains AIS. We further show NF1 loss promotes AKT-dependent transformation of fallopian tube epithelial cells and its functional restoration reinstates a p53-independent senescence-like phenotype.

## Methods and materials

### Cell lines

BJ-TERT-immortalized human foreskin fibroblasts were a gift from Robert Weinberg (Massachusetts Institute of Technology) [24]. Primary IMR-90 lung fibroblasts originating from the American Type Culture Center (ATCC CCL-186) were obtained from the Garvan Institute of Medical Research. The human fallopian tube epithelial cell line FT282, which is telomerase reverse transcriptase (TERT)-immortalized and overexpresses mutant p53-R175H [25], was generated by Ronny Drapkin and colleagues (University of Pennsylvania) and provided to us. Human embryonic kidney (HEK293T) cells were purchased from the ATCC (ATCC-CRL-3216). All cells were tested for mycoplasma contamination prior to experimentation and intermittently tested thereafter by PCR. BJ-TERT cells were cultured in Dulbecco’s Modified Eagle’s Medium (DMEM) (Gibco^™^ #11965084) +20 mM HEPES, 17% Medium 199 (Gibco^™^ #11150067), 15% fetal bovine serum (FBS), and 1% GlutaMAX^™^ l-alanyl-l-glutamine dipeptide (Gibco^™^ #35050061). IMR-90 cells were cultured in Eagle’s minimum essential medium supplemented with 10% FBS, 5 mM sodium pyruvate (Gibco^™^, #11360070), 1% nonessential amino acids (Gibco^™^, #11140050), and 1% GlutaMAX^™^. HEK-293T cells were cultured in DMEM +20 mM HEPES, 10% FBS, and 1% GlutaMAX^™^. FT282 cells were cultured in DMEM:F12 (1:1) and 2% Ultroser^™^ G serum substitute (Bio-Strategy #PALS15950-017). All cell lines were maintained at 37 °C with 5% CO_2_ except for IMR-90 (3% O_2_) in a humidified incubator. Cell numbers were determined using a Z2 Coulter counter (Beckman Coulter).

### Plasmids, virus production, and transduction

pBABE-puro, pBabe-puro-HA-myrAKT1, and pBabe-puro-HRAS^G12V^ were described previously [20]. The PHAGE-PmiR-146a-GFP-PGK-puro plasmid [26] was a kind gift from Stephen Elledge and the puromycin cassette was modified by replacing with the fluorescent selectable marker mCherry. pCW57.1-4EBP1_4xAla was deposited by David Sabatani (Addgene 38240) and HA-myrAKT1 and HRAS^G12V^ were directly subcloned into this vector to generate pCW57.1-myrAKT1 and pCW57.1-HRAS^G12V^. The doxycycline-inducible mirE small hairpin RNA (shRNA) expression vector REBIR was modified by Sang-Kyu Kim (Peter MacCallum Cancer Center) from the original TRMPVIR plasmid [27] by substituting Venus with enhanced blue fluorescent protein (EBFP2). 97-mer shRNA sequences are listed in Supplementary Table S11. Out of the three to four hairpins tested for functional knockdown in BJ-TERT cells, the two best shRNA hairpins were subcloned into the REBIR construct. Control (REN) and NF1 #2 shRNA hairpins were subcloned into the pGIPZ lentiviral shRNA plasmid (Dharmacon^™^). HEK293T cells were used for virus production and viral transductions were performed as previously described [20]. BJ-TERT cells retrovirally transduced with REBIR shRNA were isolated by FACS, sorting for EBFP2^+^/dsRED2^+^ cells after 72 h incubation with 2 μg/mL doxycycline (Sigma-Aldrich^®^ #D9891). Each cell line underwent three consecutive rounds of sorting. The REBIR plasmid was modified to generate an inducible overexpression construct RT3-puro by excising the dsRed2/mirE cassette and replacing EBFP2 with the puromycin resistance gene. FLAG-tagged NF1 GTPase activating protein-related domain (GRD) corresponding to amino acids 1194-5131 amplified from R777-E139 Hs.NF1, which was a gift from Dominic Esposito (Addgene 70423), was subcloned into RT3-puro downstream of the TRE3G promoter. Cells were selected for using 1 µg/mL puromycin. FT282 cells expressing RT3-puro-NF1-GRD were single cell cloned and expanded due to puromycin cross-resistance. MSCV-Cherry construct harboring HA-myrAKT1 has been previously described [20].

### Gas chromatography mass spectrometry

Cells were plated in six-well plates at 2 × 10^4^ cells/well density four days before harvest. Prior to harvesting, cells were washed with 3 mL 37 °C saline and treated with 3 mL liquid nitrogen until boiled off. Metabolite samples were harvested using ice-cold methanol:chloroform:scyllo-inositol (MeOH:CHCl_3_ 9:1 [v/v], 3 µM scyllo-inositol as internal standard) solution. Samples were transferred to 1.5-mL tubes and centrifuged to create an upper aqueous (MeOH) and lower organic layers (CHCl_3_). Supernatant was transferred to new tubes and snap frozen. For gas chromatography mass spectrometry (GC-MS) analysis, 300 µL of the aqueous phase was evaporated to dryness under vacuum at 4 °C. Samples were derivatized with 25 µL methoxyamine (30 mg/ml in pyridine, Sigma^®^ #226904) for 60 min at 50 °C with mixing at 900 rpm, followed by trimethylsilylation with 25 µL BSTFA + 1% TMCS (Thermo Scientific^™^ #TS-88530) for 60 min at 50 °C with mixing at 900 rpm. The derivatized sample (1 µL) was injected onto a Shimadzu GC/MS-TQ8040 system. Metabolites were normalized based on pooled metabolite peak area median and identified based on GC retention time and mass spectra as compared with authentic standards and in conjunction with MSD ChemStation Data Analysis Application (Agilent) using in-house and Wiley metabolite libraries. Statistical analyses were performed using Student’s *t* test. Metabolites were considered to be significant if their adjusted *p*-values after Benjamini and Hochberg (BH) correction were less than 0.05. Further data analysis and enrichment analysis were performed through MetaboAnalyst 4.0.

### Functional genomics RNAi screen

Full detailed methods for the functional genomics AIS escape screen are published in [28]. BJ-TERT cells were retrovirally transduced with myristoylated AKT1 (myrAKT1), establishing a senescent cell population, as we utilized for RNA-sequencing (RNA-seq) analysis. At 6 days post transduction (dpt), AIS cells were reverse transfected in 384-well plates using the siGENOME^®^ SMARTpool library to knock down 18,120 genes in an annotated, well-by-well format. RNA-seq of BJ-TERT fibroblasts (see below *RNA sequencing and analysis*) was used to filter 3,828 genes not expressed based on a raw count of 0 from three biological replicates, and were thus excluded to eliminate false positives. Seed analysis of the siRNAs corresponding to the 838 hits demonstrated minimal off-target siRNA effects. Gene ontology analysis of the primary screen hits was performed using MetaCore^™^ from GeneGo (Thomson Reuters) with functional processes associated with the top upregulated genes having a robust *Z*-score > 2. Gene network analysis of the hits duplex screen was performed with NetworkAnalyst (www.networkanalyst.ca/). Network was generated using the protein–protein interaction database from the STRING interactome with a confidence score cutoff of 900 and illustrated using Inkscape 0.92.2 software.

### siRNA transfection of IMR-90 fibroblasts

IMR-90 cells transduced with pBabe-puro empty vector control or pBabe-puro-myrAKT1 were trypsinized and counted at 6 dpt and reverse transfected in 12-well plates (for western immunoblotting) or 96-well black polystyrene clear flat bottomed plates (Corning^®^ #3603) for staining at a density of 1.5 × 10^4^ cells/cm^2^ with 0.375 μL Dharmafect 1/cm^2^ and 20 nM SMARTpool siRNA (Dharmacon^™^).

### SA-ßGal staining and EdU labeling

Ten µM EdU (5-ethyl-2′deoxyuridine) was spiked into culture media for 24 h prior to cell fixation with 2% paraformaldehyde/0.2% glutaraldehyde. Histochemical SA-ßGal staining was performed as described previously [29] for 24 h. Cells were permeabilized with 0.5% TritonX-100 in PBS, and EdU was fluorescently labeled using the Click-iT^™^ EdU Alexa Fluor^™^ 488 imaging kit (Invitrogen^™^ #C10337) as per the manufacturer’s instructions. Finally, nuclei were counterstained with 500 ng/mL 4′,6-diamidino-2-phenylindole (DAPI) in PBS. Ten random fields were imaged with an Olympus BX61 or Zeiss Axio Vert.A1 light microscope using a 20× objective. More than 100 nuclei were manually counted and the percent SA-ßGal and EdU-positive cells were quantified.

### Western immunoblotting

Whole cell lysates were prepared in western solubilization buffer as previously described [20]. Protein was transferred to PVDF membranes, which were blocked in 5% skim-milk TBS 0.1% Tween^®^ 20 (TBST) for 45 min at RT. Membranes were incubated with primary antibodies overnight (4 °C), washed three times in TBST for 10 min, incubated with HRP-conjugated secondary antibodies for 1 h at room temperature, and then washed. Membranes were visualized using Western Lightning^™^ Plus enhanced chemiluminescence (Perkin Elmer) by exposure to film (Fujifilm SuperRX) or imaged by a ChemiDoc^™^ Touch Imaging System (Bio-Rad Technology). Digital scans of film were acquired using an Epson Perfection V700 Photo at ≥300 dpi. A list of antibodies used is available in Supplementary Table S12. Ras activity was assessed using the Active Ras Detection Kit (Cell Signaling Technology^®^ #8821) according to the manufacturer’s instructions.

### Gene expression analysis

Total RNA was extracted using the ISOLATE-II (Bioline) or RNeasy^®^ Mini Kit (Qiagen) as per the manufacturer’s instructions. One μg of total RNA was used as a template for cDNA synthesis using SuperScript^™^ III reverse transcriptase (Invitrogen^™^ #18080093), hexameric random primers, and dNTPs. Quantitative real-time PCR (qRT-PCR) reactions were performed using Fast SYBR^®^ Green reagents in a StepOnePlus^™^ Real-Time PCR system (Applied Biosystems^™^) with a +0.7 °C melt increment. Changes in target gene expression were normalized to *GAPDH* housekeeping genes and fold change was determined by using 2^(−ΔΔ*Ct*). Primer sequences are listed in Supplementary Table S13.

### RNA-sequencing and analysis

Poly-A selective RNA-seq libraries were prepared using the TruSeq RNA sample preparation kit (Illumina^®^) and sequenced on an Illumina^®^ Genome Analyzer IIx (pool of six samples per lane) or Illumina^®^ NextSeq 500. The quality of the generated 50 bp paired-end reads from BJ-TERT cells (AIS versus proliferating) was assessed with FastQC 0.11.2. Tophat2 (version 2.0.13)/Bowtie2 (version 2.2.3.0) or HISAT2 (version 2.0.4) for the 75 bp single reads (OIS versus pBabe) [30], which were used for alignment to the genome (hg19/GRCh37). Cutadapt (version 1.7.1 with Python 2.7.8) was used for adapter and quality trimming. Picard 1.125 was used to generate BAM indices and to calculate insert size. Samtools 0.1.18 was used to sort and merge. Reads were counted using featurecounts (version 1.6.2) in Galaxy [31]. The differential expression of genes was then calculated utilizing the DESeq2 package v1.24.0 and plotted in R [32]. Absolute gene expression was defined determining RPKM as previously described [33]. FastQ raw data and processed files are available in the public depository NCBI GEO under accession numbers GSE130099 and GSE130100. Gene set enrichment analysis (GSEA) of a preranked list log_2_FC × −log_10_(*p*-value) with average raw counts ≥20 from three biological replicates from at least one sample set was performed according to the Hallmark gene set from the molecular signatures database MSigDBv6.1 (Broad Institute). Heatmaps were generated using Morpheus (Broad Institute).

### Cytokine array

For antibody arrays, 5 × 10^5^ were cultured in six-well plates for 72 h prior to replacing defined medium with 0.5 mL of serum-free DMEM for 24 h. Conditioned medium was collected and clarified by centrifugation at 2000 *g* for 10 min. The supernatant was diluted and applied to the Proteome Profiler^™^ Human Cytokine Array Kit (R&D Systems^®^ ARY005B). Spot intensity was quantified with the Protein Array Analyzer ImageJ Plugin (http://image.bio.methods.free.fr/ImageJ/?Protein-Array-Analyzer-for-ImageJ.html) and normalized to cell number as previously described [14].

### Clonogenic assays

Clonogenesis was assessed as previously described [34]. Cells were fixed in 100% methanol and stained with 0.1% crystal violet. Representative images were acquired using a Bio-Rad Chemidoc^™^ Touch Imaging platform. Colonies were counted manually and determined as positive if they contained at least 50 cells [35]. Percent area and stain intensity were determined using the ImageJ plugin ColonyArea [35]. Colony area is a measure of well coverage and intensity provides an indication of the number of cells per colony.

### Anchorage-independent growth

The soft agar assay for anchorage-independent growth was performed as in [36] with modifications. A base layer of 1.5 mL of 0.6% Difco^™^ noble agar (BD Biosciences #214230) in defined growth medium was applied to six-well plates and allowed to set at room temperature. Cells were then suspended in 1.5 mL of 0.4% agar and seeded in triplicate for each independent experiment. Colonies were fed 100 μL media twice weekly for 28 days. A total of 20 random fields were visualized using an EVOS^™^ FL Cell Imaging System (Thermo Scientific^™^) and colonies (>50 μm in diameter) were manually counted.

### Three-dimensional (3D) spheroid culture

A base layer of 100 µL 1.5% Difco^™^ noble agar solution in PBS was applied to a flat-bottomed 96-well plate and allowed to solidify at room temperature. FT282 cells were resuspended at 10^4^ cells/mL, 100 µL cell suspension was seeded each well in FT282 media, and spheroids were allowed to develop for 4 days. Spheroids were then embedded in a 20 µL droplet of Matrigel^®^ Growth Factor Reduced Basement Membrane Matrix (Corning^®^ #356231) in a 48-well flat-bottom polystyrene tissue culture plate (Corning^®^ #3548). The spheroids in Matrigel^®^ were overlaid with 250 µL FT282 media without or with 1 µg/mL doxycycline for 3 days and replenished with fresh media for additional 2 days, followed by incubation with 10 µM EdU for 24 h. Matrigel^®^ was depolymerized with Cell Recovery Solution (Corning^®^ #354253) for 30 min at 4 °C. Spheroids were washed twice with PBS and fixed in 2% paraformaldehyde + 0.2% glutaraldehyde for 10 min for immunohistochemistry analysis. Fixed spheroids were embedded in Richard-Allan Scientific^™^ HistoGel^™^ Specimen Processing Gel (Thermo Scientific^™^ #HG-4000-012), paraffinized, and 4-µm sections were mounted onto glass slides. Sections were dewaxed, permeabilized with 0.5% TritonX-100 in PBS for 5 min, and stained using the Click-iT^™^ EdU Alexa Fluor^™^ 647 Imaging Kit (Invitrogen^™^ C10340) as per the manufacturer’s instructions. DAPI staining was used to label cell nuclei. Slides were imaged with an Olympus BX61 using a 20× or 40× objective. The number of cells per spheroid and EdU-positive cells were manually counted.

### Clonality/subclonality ratio analysis of TRACERx nonsmall lung cancer data

The raw data from the TRAcking Cancer Evolution through therapy (Rx) (TRACERx) study are available at the European Genome-phenome Archive under accession number (EGAS00001002247). There was a total of 99 lung nonsmall cell samples used by the repeated evolution in cancer (REVOLVER) R Package [37] to determine whether mutations were clonal or subclonal. The bioinformatics analyses to obtain somatic alterations and cancer cell fractions (CCFs) were performed by TRACERx. Raw paired end reads (100 bp) were aligned to the hg19 genomic assembly using bwa mem (bwa-0.7.7). Picard tools v1.107 was used to clean, sort, and merge files from the same patient region and to remove duplicate reads (http://broadinstitute.github.io/picard). VarScan2 somatic (v2.3.6) and MuTect (1.1.4) were used to identify variants. SNV with a variant allele frequency (VAF) > 2% and called by both VarScan2 and MuTect, or a VAF > 5% if only called by VarScan2 were used for downstream analysis. CCFs, which is the proportion of cancer cells in a sample with a particular alteration, were determined using PyClone. REVOLVER uses the CCFs as input. In REVOLVER, an alteration could be of any type, including single-nucleotide variants, copy-number alterations, or epigenomics events. The analysis software for REVOLVER, as well as the data of patients, of CCFs and whether a variant was clonal or subclonal is available at https://github.com/caravagn/revolver. The clonality/subclonality ratios were derived from the information provided by the REVOLVER package of whether mutations were clonal or subclonal. We obtained the clonality of mutations of lung (TRACERx) cohorts, where patient-level trees capturing the evolution of the cancer were derived based on multiregion sequencing using machine learning based on a transfer learning method [37, 38]. We only considered the 36 AIS escape screen genes with mutations in ≥3 patients and were clonal or subclonal in ≥1 patient. When multiple mutations occurred in the same gene in the same patient, we considered these a single event. We calculated a clonality/subclonality ratio for each gene *G* as follows:

$$	{\rm{Clonality}}/{\rm{subclonality}}\,{\rm{ratio}}_{G} \\ 	= \frac{ {\rm{Number}}\,{\rm{of}}\,{\rm{patients}}\,{\rm{with}}\,{\rm{a}}\,{\rm{clonal}}\,{\rm{mutation}}\,{\rm{in}}\,G}{{\rm{Number}}\,{\rm{of}}\,{\rm{patients}}\,{\rm{with}}\,{\rm{a}}\,{\rm{subclonal}}\,{\rm{mutation}}\,{\rm{in}}\,G}$$

### Single-sample gene set enrichment analysis (ssGSEA) of The Cancer Genome Atlas (TCGA) data

We examined the TCGA dataset using cBioportal [39, 40] and selected patients only with RNASeq (v2) gene expression, mutation, and copy number data, resulting in an initial pool of 5,331 samples belonging to 14 cancer types (bladder urothelial carcinoma; breast adenocarcinoma; cervical and endocervical cancers; glioblastoma multiforme; head and neck squamous cell carcinoma; lung adenocarcinoma; lung squamous cell carcinoma, LUSC; ovarian serous cystadenocarcinoma; prostate adenocarcinoma; sarcoma; stomach adenocarcinoma; thyroid carcinoma; uterine corpus endometrial carcinoma; uterine carcinosarcoma). Of these samples, 19 patients had cell cycle and apoptosis regulator 1 (*CCAR1*) mutations, 5 had Fas-associated protein with death domain (*FADD*) mutations, and 21 had *NF1* mutations. Two LUSC patients had *CCAR1* mutations and three had *NF1* mutations. We derived an AIS escape signature using the 98 genes identified from the AIS escape screen. In the LUSC with *CCAR1* or *NF1* mutations, we calculated the level of expression of the AIS escape signature in individual patients using ssGSEA [41] from the GSVA R package [42] on the RNA-seq data. For the reference group, we only considered patients with mutation, copy-number aberration, and gene expression data to ensure the group excluded patients with alterations in *CCAR1*, *FADD,* or *NF1*. One-sided Wilcoxon rank sum tests were used to test for statistical significance.

### Code availability

R code repository for this manuscript is available at https://github.com/keefe-chan/AKT-induced-senescence-functional-genetic-screen.

### Statistical analyses

Pooled data are presented as mean ± SEM values for triplicate biological replicates as indicated in the figure legends. Data were presented using GraphPad Prism (8.1) and *P*-values < 0.05 were considered significant. Statistical analyses were not used to determine sample size.

## Results

### Transcriptomic and metabolic profiling identify key AIS signatures

**Fig. 1:**
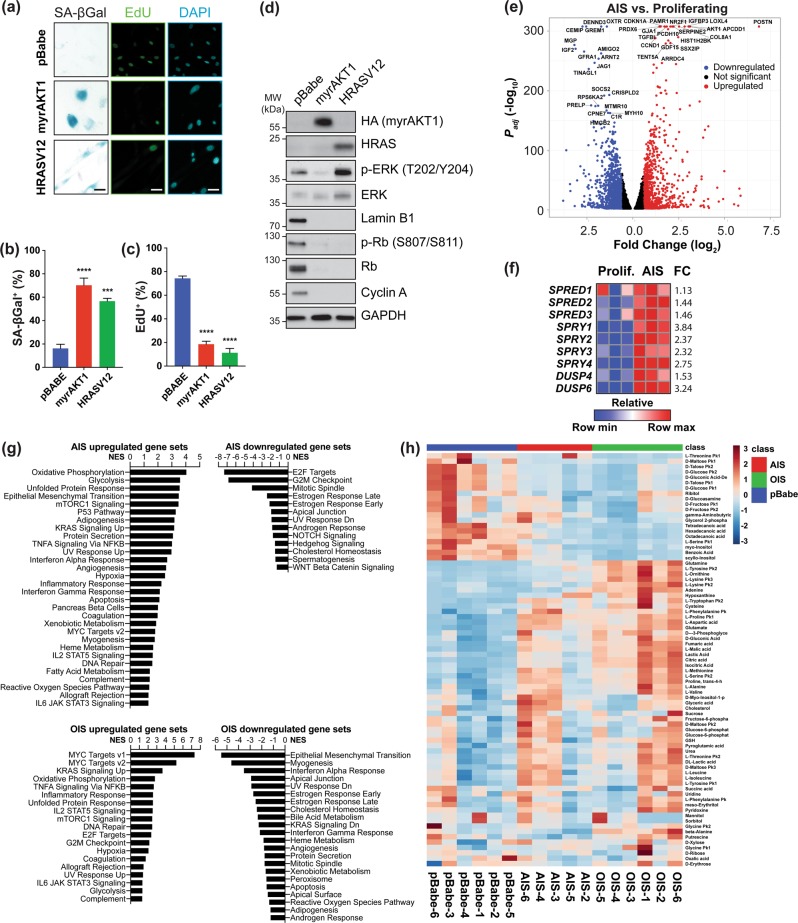
Transcriptomic and metabolic profiling identify signatures of AIS. **a–d** BJ-TERT cells were transduced with pBabe empty vector control, myrAKT1, or HRASV12 and analyzed at 14 dpt. **a** Cells were stained for SA-ßGal activity or EdU. DAPI staining was used to visualize nuclei. Scale bars = 50 μm. **b**, **c** Quantification of percentage of cells with positive staining for **b** SA-ßGal activity or **c** EdU. **d** Western blots showing senescence markers as well as phosphorylated and total ERK. GAPDH was probed as a loading control. **e** Differentially expressed genes (FC ≤ −1.5 or FC ≥ 1.5, FDR ≤ 0.01) between cells undergoing AIS versus proliferating cells, which are downregulated (blue), upregulated (red), or not significant (black). The top 20 significantly upregulated and downregulated genes are indicated. **f** Heatmap from RNA-seq data of AIS versus proliferating cells showing upregulation and FC of inhibitors of RAS/ERK signaling. *n* = 3 biological replicates. **g** Gene set enrichment analysis showing normalized enrichment score (NES) for significantly upregulated and downregulated gene sets during AIS or OIS compared to proliferating cells. **h** Hierarchical clustering of the top 50 significant metabolites in proliferating cells or those undergoing AIS or OIS. Note: The sample AIS-1 was excluded due to a technical issue in sample processing independent of sample quality

We previously showed normal human cells with chronic PI3K/AKT/mTORC1 or RAS/ERK activation display numerous senescence markers [20]. We confirmed constitutively activating either pathway increased SA-ßGal-positive, decreased EdU-positive cells (Fig. 1a–c), and decreased levels of the cell cycle regulators phosphorylated and total Retinoblastoma protein (Rb) and cyclin A (Fig. 1d). Numerous prosenescence stimuli induce microRNA (miR)-146a expression [26]. To test if this occurs during AIS, we used an miR-146a promoter-GFP fusion construct coupled to a constitutive Cherry reporter (Fig. S1a). FACS analysis at 14 days post transduction (dpt) showed increased miR-146a expression during AIS (Fig. S1b, c), indicating miR-146a upregulation is also common to AIS and OIS.

Previously, we showed p53 mediates proliferative arrest during AIS, while p16 prevails during OIS, suggesting a different set of mediators governs each phenotype [20]. Although several OIS mediators are known, those for AIS are not. To understand and identify whether these mechanisms are distinct from OIS, we transcriptionally profiled proliferating TERT-immortalized human fibroblasts (BJ-TERT) and those expressing myrAKT1, a PI3K/AKT/mTORC1 pathway hyperactivation surrogate, using RNA-seq (Table S1). At 6 dpt [20], 1003 genes were upregulated (fold change (FC) ≥ 1.5, FDR < 0.01) and 1350 genes were downregulated (FC ≤ −1.5, FDR ≤ 0.01) compared to control, highlighting significant transcriptional rewiring during AIS (Fig. 1e). *CDKN1A*, encoding the cyclin-dependent kinase inhibitor p21, increased >3-fold, consistent with p53 engagement [20]. *LMNB1*, encoding lamin B1 was also downregulated (Fig. 1d), agreeing with impaired nuclear integrity during senescence contributing to chromatin reorganization and gene expression changes [43]. We also observed robust SASP-related gene expression (Fig. S1d) [14], and qRT-PCR at 14 dpt confirmed *CXCL1*, *IL1A*, and *IL1B* induction (Fig. S1e–g).

To further distinguish AIS from OIS, we performed RNA-seq of BJ-TERT fibroblasts expressing constitutively active HRASV12 at 14 dpt (Table S2), when both had a similar percentage of senescent cells (Fig. 1a–c). Contrasting AIS, OIS did not show increased *CDKN1A* expression, but showed a >2-fold increase over control in *CDKN2A*, encoding for p16. We also observed downregulated *LMNB1* mRNA (Table S2) and lamin B1 protein (Fig. 1d), and increased SASP-related gene expression (Fig. S1d). However, SASP gene expression between OIS and AIS did not overlap completely, as IGFBP3, IGFBP5, IGBFP7, and FN1 expression decreased and CXCL3 markedly increased during OIS compared to AIS.

RNA-seq analysis also revealed RAS/ERK pathway inhibitor upregulation during AIS: the Sprouty-related genes *SPRED1*, *SPRED2*, and *SPRED3*; Sproutys *SPRY1*, *SPRY2*, *SPRY3*, and *SPRY4*; and the ERK-inactivating dual specificity phosphatases *DUSP4* and *DUSP6* (Fig. 1f). This concurs with negative feedback signaling during OIS [44] and indicates transcriptional induction of negative regulators of RAS/ERK signaling is common to AIS and OIS. However, AIS distinctly shows diminished RAS/ERK signaling at the protein level, while it is robustly increased during OIS (Fig. 1c).

To further dissect the critical pathways regulating AIS or OIS, we performed GSEA [45], demonstrating common and distinct AIS and OIS hallmarks. Both displayed upregulated oxidative phosphorylation, glycolysis, unfolded protein response, mTORC1 signaling, and TNFα signaling via NF-κB (Fig. 1g; Table S3); and downregulated estrogen response, mitotic spindle, apical junction, UV response, and cholesterol homeostasis (Fig. 1g; Table S4).

In contrast, interferon response and epithelial mesenchymal transition gene sets were enriched during AIS and downregulated during OIS. Diminished interferon response during OIS agrees with antagonism by p38 pathway activation [46]. Also, E2F targets and G_2_M checkpoint gene sets were downregulated during AIS and upregulated during OIS, reflecting the distinct mechanisms for engaging p53 and p16 [20]. Collectively, these data demonstrate constitutive PI3K/AKT/mTORC1 or RAS/ERK pathway activation in nontransformed cells evokes overt cell cycle arrest and inflammatory phenotypes, however important subtleties underlie how each drives its overarching senescence phenotype.

Another critical OIS feature is markedly altered metabolic activity, featuring decreased glycolysis, increased lipid content, impaired mitochondrial function, and increased oxidant formation compared to proliferating cells [47]. To understand whether similar metabolic changes occurred during AIS, we profiled proliferating cells and those undergoing AIS or OIS using GC-MS (Table S5). Principal component (Fig. S2a) and hierarchical clustering (Fig. 1h) analyses showed clear separation between the metabolites generated by proliferating cells versus those undergoing AIS or OIS. Of the significant metabolites (BH adjusted <0.05), 27 were common to AIS or OIS (Fig. S2b). Six metabolites (glutamate, cysteine, glutamine, glyceric acid, ribitol, and proline.trans.4.hydroxyl.L) were specifically upregulated during AIS. Metabolic pathway enrichment analysis also demonstrated AIS and OIS had enriched urea cycle, Warburg effect, glycine and serine metabolism, malate-aspartate shuttle, and phenylalanine and tyrosine metabolism pathways compared to proliferating cells (Fig. S2c, d and Table S6). These pathways were more significantly enriched during OIS, possibly reflecting how the DDR reinforces senescence-associated metabolic rewiring [47]. Together with our previous findings, these transcriptomics and metabolomics data demonstrate AIS and OIS have common and distinct molecular underpinnings.

### A genome-wide RNAi screen identifies novel AIS mediators

**Fig. 2:**
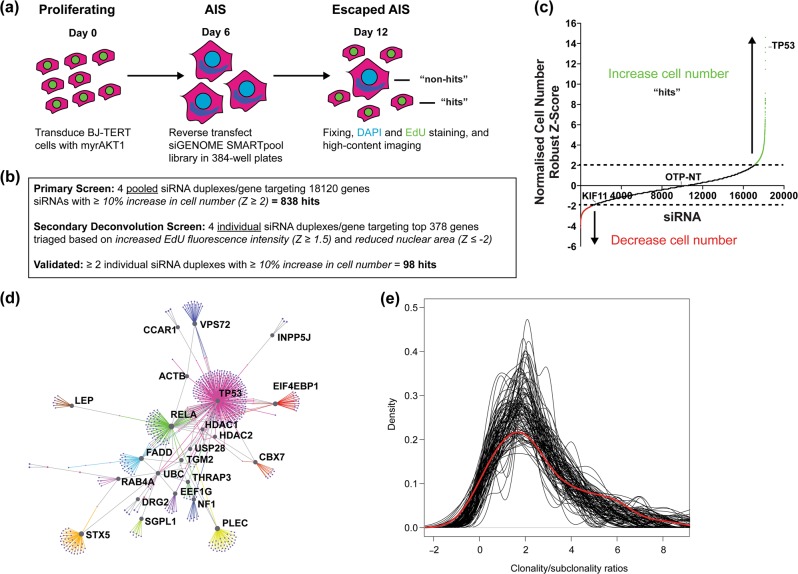
A genome-wide RNAi screen reveals critical regulators of AIS maintenance. **a** Schematic representation of screen design. BJ-TERT fibroblasts were retrovirally transduced with constitutively active myristoylated AKT1. At 3 dpt, senescent cells were enriched by puromycin selection for 3 days. At 6 dpt, senescent cells were reverse transfected in arrayed format with SMARTpool siRNAs in 384-well plates. At 12 dpt (6 days post transfection), cells were fixed and stained for SA-ßGal activity and EdU for proliferation. DAPI staining was used to label cell nuclei for quantification of cell number. **b** Summary of screen results. Hits were determined based on statistical robust *Z*-score cutoffs for cell number, average EdU fluorescence intensity, and nuclear area normalized to nontargeting siRNA (siOTP-NT). RNA-seq of BJ-TERT cells (Supplementary Table S1) was used to exclude 3828 genes not expressed to minimize false positives. **c** Plot of siRNA target versus robust *Z*-score for cell number normalized to nonsilencing control in the primary screen as indicated in **b**. **d** First-order network generated from secondary deconvolution screen hits as indicated in **b**. **e** Regulators of AIS maintenance are enriched during the clonal evolution of human lung cancer. Plot of clonality/subclonality ratios from the screen dataset (red) versus 100 out of 100,000 random gene set distributions (black)

Multiple OIS mediators have been uncovered [48–50]. To determine the essential AIS regulators and identify mechanisms overcoming AIS, we assayed AIS escape using high-throughput microscopy [28] in a genome-wide RNAi screen of BJ-TERT cells retrovirally transduced with myrAKT1 (Fig. 2a, b; Table S6). Tumor protein p53 (*TP53*) siRNA was used as an AIS escape positive control. At 12 dpt (6 days post transfection), we quantified cell number, EdU incorporation, and nuclear area (Table S7). Senescent cells were fewer in number and had larger nuclei due to altered nuclear integrity and chromatin reorganization [43]. Positive hits restoring cell proliferation in the primary screen were siRNAs with a robust *Z*-score scoring ≥2 standard deviations above the plate mean (≥10% increased cell number) (Fig. 2b, c). Gene ontology analysis of the primary screen data revealed functional processes including responses to compounds and lipids, regulation of cell communication, and positive regulation of NF-κB signaling (Table S8).

We triaged the 838 hits based on two parameters: average EdU fluorescence intensity (robust *Z*-score ≥ 1.5, ≥17% increase) and nuclear area (robust *Z*-score ≤ −2, ≥12% decrease), as proliferating cells would show restoration of these markers. The top 378 hits were assessed in secondary screening, where SMARTpools were deconvoluted into the four individual siRNA duplexes. Genes with ≥2 siRNAs recapitulating the SMARTpool phenotype were considered hits (Table S9). Gene network analysis of the 98 hits identified *TP53* (the top hit), and v-rel avian reticuloendotheliosis viral oncogene homolog A (*RELA*) as nodes displaying the highest degree and betweenness (Fig. 2d and Table S10), indicating they integrate most signaling inputs within the AIS network. Also, 10% of the hits were associated with cellular metabolism (*SLC15A2*, *SLC25A13*, *SLC7A6*, *SLC20A1*, *SLC7A2*, *GGH*, *SLC22A11*, *NOX5*, *NOXO1*, *CBS*, and *CTNS*), suggesting extensive metabolic reprogramming during AIS, consistent with our metabolomics analysis. Inositol 5-phosphatase *INPP5J*, a screen hit and AKT negative regulator, was a recently identified breast cancer suppressor [51], supporting our screen’s robustness.

Given the screen hits maintain AIS, their loss or inactivation may occur during PI3K/AKT/mTORC1-driven tumorigenesis. To test this concept, we interrogated the TRACERx study, which utilizes multiregion exome sequencing to determine clonal evolutionary processes during tumor development in human nonsmall cell lung cancer of which 50–70% display aberrant PI3K/AKT/mTORC1 activation [37, 38]. Mutations were classified as clonal (present in all cells), occurring early during tumor evolution, or subclonal (present in a subset), occurring later. To calculate whether the AIS escape screen genes had higher clonality/subclonality ratios than those not in the screen, we subsampled 100,000× gene sets containing 36 random genes (Fig. 2e). While the actual distribution’s average clonality/subclonality ratio was 3.06, the random distributions’ was 2.52. Comparing the skewness of the screen and random distributions, we empirically derived a permutation *P-*value = 0.03, suggesting mutations in the AIS escape screen genes tend to be clonal than other genes. The genes with significant clonality/subclonality ratios were *TP53, PTPRZ1, DENND5, KLHL17, MOCOS, and TGM2*, highlighting the potential contribution of these AIS mediators to the early development of lung cancer.

### RELA regulates AIS in BJ-TERT cells

During OIS the NF-κB pathway drives inflammatory signaling [52, 53]. Although *TP53* and *RELA*, encoding the p65 subunit of the canonical NF-κB signaling complex, are the AIS network’s central nodes, NF-κB activation during AIS has not been evaluated. To test this, we generated BJ-TERT cell lines expressing inducible shRNAs (Fig. S3a) against *RELA* and *TP53* as a positive control, enabling longer-term functional studies. After transducing BJ-TERT cells with myrAKT1 or HRASV12 promoting AIS or OIS, we induced control or *RELA* knockdown with doxycycline (Fig. S3b) and confirmed p65 knockdown (Fig. S3c). To test AIS stability and whether *RELA* depletion could overcome AIS, we stained for SA-ßGal activity and EdU at 19 dpt (Fig. S3d). While cells during AIS or OIS were EdU-negative and SA-ßGal positive, *RELA* knockdown decreased SA-ßGal-positive and increased EdU-positive cells, indicating cell cycle re-entry (Fig. S3e, f). To further validate the AIS-escaping cells’ proliferative potential upon *RELA* depletion, we performed clonogenic assays, demonstrating enhanced colony formation (Fig. S3g–i).

To test *RELA* knockdown’s impact on the SASP, we examined conditioned medium on cytokine membrane arrays (Fig. S3j). CXCL1, G-CSF, IL-6, and IL-8 were upregulated during AIS and OIS (as a positive control) compared to proliferating cells. *RELA* knockdown in myrAKT1-expressing cells decreased G-CSF and IL-6 but not CXCL1 or IL-8, indicating p65 regulates a subset of SASP cytokine production during AIS in BJ-TERT cells (Fig. S3j, k).

### CCAR1, FADD, and NF1 are novel AIS mediators

We next investigated AIS escape screen mediators involved in tumor suppression but not yet implicated in regulating senescence. CCAR1 expression is reduced in poorly differentiated breast cancers and FADD expression is downregulated during thyroid adenocarcinoma progression, with both cancers showing aberrant PI3K/AKT/mTORC1 pathway activation [54, 55]. The tumor suppressor NF1, the second-ranked hit behind p53 (Table S9), is implicated in melanoma though its role in AIS is unknown [18, 44, 56].

**Fig. 3:**
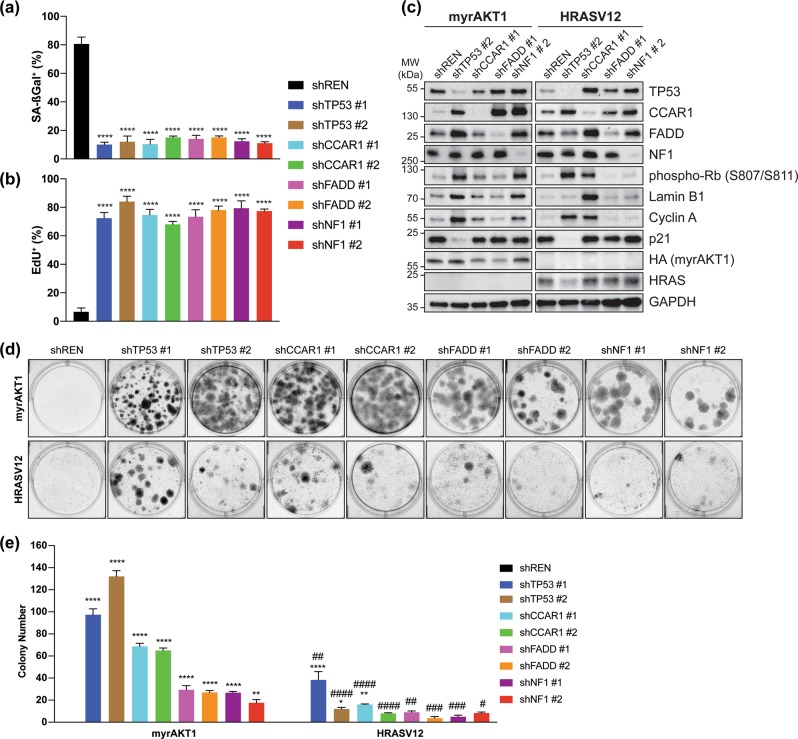
Functional validation of AIS mediators. **a–c** BJ-TERT cells expressing inducible shRNA were transduced with myrAKT1, treated with doxycycline and assessed for AIS escape by quantification of percentage of cells with positive staining for **a** SA-ßGal activity or **b** EdU, or **c** western blotting for senescence markers after 19 days. **d**, **e** BJ-TERT cells expressing inducible myrAKT1 or HRASV12 and inducible shRNA were treated with doxycycline and assessed for AIS bypass in **d** colony formation assays after 19 days. **e** Quantification of colony number in **d**. Data are expressed as mean ± SEM. *n* = 3 experiments. *P < 0.05; **P < 0.01; ****P < 0.0001 by one-way *ANOVA* as compared with shREN for each oncogene. ^###^*P* < 0.01; ^####^*P* < 0.0001 by two-way *ANOVA* as compared with myrAKT1 and corresponding shRNA

To functionally validate these mediators, we generated BJ-TERT cell lines expressing doxycycline-inducible shRNAs. At 6 dpt with myrAKT1, we induced shRNA knockdown and assessed SA-ßGal and EdU staining at 19 dpt (Fig. 3a, b). Depleting *TP53*, *CCAR1*, *FADD,* and *NF1* showed decreased SA-ßGal-positive and increased EdU-positive cells compared to control, indicating significant AIS disengagement.

To determine mediator specificity, we compared senescence signaling in these cell lines during AIS and OIS. CCAR1, FADD, and NF1 knockdown levels were equivalent between AIS and OIS (Fig. 3c). Control cells undergoing AIS or OIS had low levels of phosphorylated Rb, lamin B1, cyclin A, and p21. *TP53* knockdown increased phosphorylation of Rb, lamin B1, and cyclin A, and decreased p21 during AIS, indicating cell cycle re-entry/senescence escape. HRASV12-expressing cells with *TP53* knockdown also showed increased phosphorylated Rb and cyclin A, and decreased p21 but unlike myrAKT1-expressing cells, did not display increased lamin B1. *CCAR1* depletion increased phosphorylated Rb, lamin B1, and cyclin A in myrAKT1-expressing cells and markedly in HRASV12-expressing cells, suggesting it is a common mediator. *FADD* knockdown in myrAKT1-expressing cells increased phosphorylated Rb but not in HRASV12-expressing cells. Intriguingly, *NF1* depletion robustly increased phosphorylation of Rb, lamin B1, and cyclin A only in myrAKT1-expressing cells, indicating specificity for NF1 during AIS. Furthermore, p21 expression was unchanged with all knockdowns except for *TP53* during AIS, supporting p53-independent senescence escape mechanisms.

We further interrogated these mediators in OIS and AIS bypass using BJ-TERT cell lines coexpressing doxycycline-inducible shRNAs and inducible myrAKT1 or HRASV12. Clonogenic assays demonstrated enhanced colony formation upon knockdown of the mediators compared to control, indicating AIS bypass (Fig. 3d, e). Colony formation was increased with knockdown of these genes upon constitutive RAS/ERK activation, albeit less robust than observed for AIS, even for *TP53*, indicating the mediators tested play a more significant role in regulating AIS than OIS.

We also tested the senescence mediators in primary human diploid IMR-90 lung fibroblasts (Fig. S4a). IMR-90 cells undergoing AIS following myrAKT1 transduction stained strongly with SA-ßGal and were EdU-negative. Depleting *TP53*, *CCAR1*, *FADD*, or *NF1* in myrAKT1-expressing cells showed reduced SA-ßGal-positive and increased EdU-positive cells. In contrast, their knockdown in proliferating cells did not affect EdU or SA-ßGal staining (Fig. S4b–d). Intriguingly, unlike in BJ-TERT cells, *RELA* depletion could not cause AIS escape in IMR-90 cells, consistent with *RELA* regulation of senescence being cell type specific [57].

To further ascertain clinical relevance, we selected TCGA patients with mutations/copy number alterations in *NF1*, *FADD*, and/or *CCAR1* using cBioportal [39, 40]. We derived an AIS escape signature comprising the 98 screen hits and determined its expression in individual LUSC patients across the TCGA cohort using ssGSEA on the RNA-seq data. While no patients had *FADD* alterations in LUSC, when we compared patients with mutations in *NF1* (*n* = 3) or *CCAR1* (*n* = 2) versus those without, the AIS escape signature was enriched (*P* = 0.03) of (Fig. S5). These data demonstrate some patients with mutations in AIS mediators also have gene expression changes associated with AIS escape mechanisms.

### NF1-mediated suppression of RAS/ERK signaling maintains AIS

**Fig. 4:**
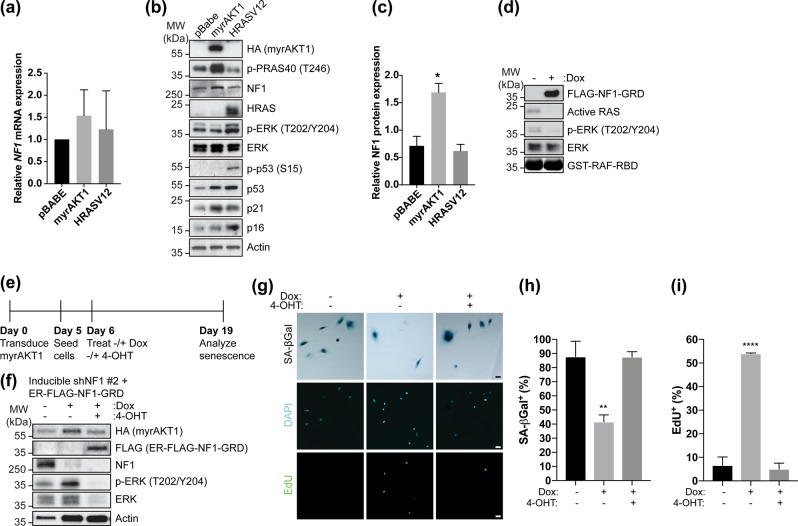
NF1-mediated suppression of RAS/ERK signaling is required for AIS. **a–c** BJ-TERT cells were transduced with pBabe, myrAKT1, or HRASV12 and analyzed after 14 days. **a** qRT-PCR showing relative *NF1* mRNA expression normalized to *GAPDH* and pBabe control. Data are expressed as mean ± SEM. *n* = 3 experiments. **b** Western blotting of NF1, PI3K/AKT/mTORC1, or RAS/ERK pathway activation and senescence signaling. Actin was probed as a loading control. **c** Quantification by densitometry of relative NF1 protein expression normalized to actin loading control. Data are expressed as mean ± SEM. *n* = 3 experiments. *P < 0.05 by one-way *ANOVA* as compared with pBabe control. **d** BJ-TERT cells expressing inducible NF1-GRD were treated without or with doxycycline for 3 days and analyzed for active RAS by western blotting. **e**–**i** BJ-TERT cells expressing inducible NF1 shRNA #2 and ER-FLAG-NF1-GRD were transduced with myrAKT1 and left untreated or treated with doxycycline at 6 dpt in the absence or presence of 40 nM 4-OHT for 13 days. **e** Experimental design. **f** Western blots showing phosphorylated and total ERK expression upon NF1 depletion and ER-FLAG-NF1-GRD expression. **g** Cells were stained for SA-ßGal activity and EdU. Scale bars = 50 μm. **h**, **i** Quantification of percentage of cells with positive staining for **h** SA-ßGal activity or **i** EdU. Data are expressed as mean ± SEM. *n* = 3 experiments. **P < 0.01; ****P < 0.0001 by one-way *ANOVA* as compared with untreated control

Our RNA-seq and protein expression analyses revealed suppression of RAS/ERK signaling is a key AIS hallmark (Fig. 1d, f). We also demonstrated NF1 loss results in AIS escape and bypass (Fig. 3a–e). Given NF1 is a RAS GTPase-activating protein [56], we hypothesized NF1-mediated suppression of RAS/ERK signaling maintains AIS. RNA-seq (Table S1) and qRT-PCR (Fig. 4a) analyses showed unchanged *NF1* mRNA expression upon AIS. However, NF1 protein expression increased twofold during AIS but not OIS compared to proliferating cells (Fig. 4b, c), coinciding with decreased phosphorylated ERK during AIS.

We tested if functional NF1 restoration could prevent AIS escape. The NF1 GTPase-activating protein-related domain (GRD) is sufficient to inactivate RAS [58] (Fig. 4d). We thus generated a 4-hydroxytamoxifen (4-OHT)-inducible estrogen receptor (ER) FLAG-tagged NF1-GRD fusion, and used a 4-OHT dose resulting in a phosphorylated ERK level comparable to that during AIS (Fig. 4e, f). BJ-TERT cells coexpressing doxycycline-inducible NF1 shRNA and 4-OHT-inducible NF1-GRD were made senescent upon myrAKT1 transduction, and were treated without or with doxycycline to induce NF1 knockdown and AIS escape. Doxycycline-treated cells were also treated without or with 4-OHT. While NF1 depletion caused AIS escape, cells with NF1 knockdown and simultaneous NF1-GRD induction remained senescent (Fig. 4g–i), indicating on-target shRNA specificity and AIS maintenance by restoring NF1-mediated suppression of RAS/ERK signaling.

### Overexpressing an NF1-GRD fragment blocks p53-mutant fallopian tube epithelial cell transformation

Given 50% of cancers harbor p53-inactivating alterations we investigated whether we could exploit the screen candidates to reinstate AIS in cells with hyperactive AKT and p53 deficiency. The fallopian tube secretory epithelial cell line FT282 [25] models the premalignant precursor to high-grade serous ovarian cancers (HGSOC), of which 96% harbor *TP53* mutations and >35% display constitutive PI3K/AKT/mTORC1 pathway activation [59]. Furthermore, 20% harbor *NF1*-inactivating mutations and/or gene breakage [60, 61], supporting a cooperative role for NF1 loss in HGSOC.

**Fig. 5:**
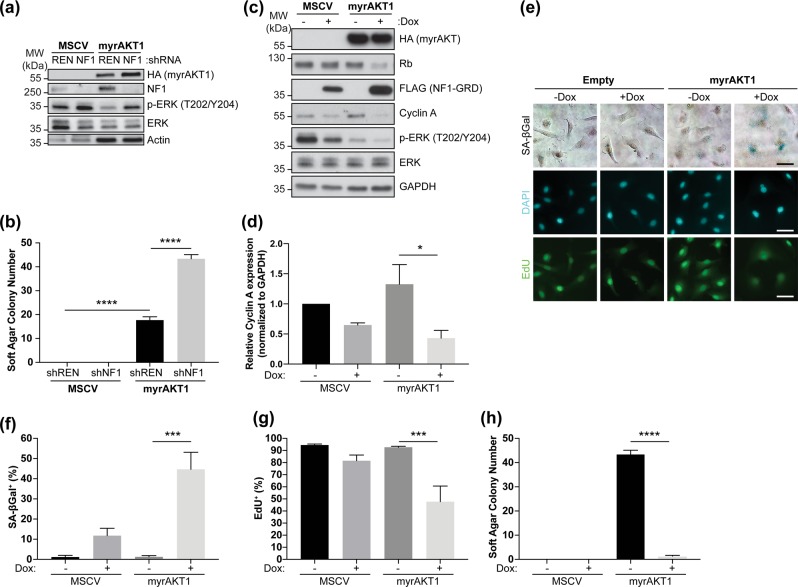
Overexpression of an NF1-GRD fragment promotes an AIS-like phenotype in p53-mutant fallopian tube epithelial cells. **a** Western blots showing NF1 and phosphorylated and total ERK expression in FT282 cells expressing MSCV empty vector control or myrAKT1 and control (REN) or NF1 shRNA #2. Actin was probed as loading control. **b** Quantification of soft agar colony number from anchorage-independent growth assays after 28 days for FT282 cells expressing MSCV empty vector control or myrAKT1 and control (REN) or NF1 shRNA #2. Data are expressed as mean ± SEM. *n* = 3 experiments. ****P < 0.0001 by one-way *ANOVA* as compared with shREN. **c–h** FT282 cells depleted of NF1 (shNF1 #2) expressing inducible NF1-GRD and MSCV empty vector control or myrAKT1 were treated without or with doxycycline. **c** Western blotting for markers of proliferative arrest after 3 days. GAPDH was probed as a loading control. **d** Quantification of relative cyclin A expression normalized to GAPDH in **c**. **e** Cells were stained for SA-ßGal activity or EdU after 6 days. DAPI staining was used to visualize nuclei. Scale bars = 50 μm. **f**, **g** Quantification of percentage of cells with positive staining for **f** SA-ßGal activity or **g** EdU. **h** Quantification of soft agar colony number from anchorage-independent growth assays after 28 days. Data are expressed as mean ± SEM. *n* = 3 experiments. ***P < 0.001; ****P < 0.0001 by one-way *ANOVA* as compared with empty vector control—Dox

We generated isogenic control or myrAKT1-expressing FT282 cells and confirmed functional expression (Fig. 5a). Anchorage-independent growth assays demonstrated myrAKT1 could transform the FT282 cells (Fig. 5b), while NF1 knockdown alone was insufficient. However, NF1 loss increased soft agar colony formation in FT282 myrAKT1-expressing cells, indicating cooperation with PI3K/AKT/mTORC1 activation and mutant p53 to drive transformation (Fig. 5b).

NF1-deficient FT282 cells showed increased phosphorylated ERK, which was reduced upon myrAKT1 expression (Fig. 5c), supporting our observation in BJ-TERT cells that hyperactive AKT induces feedback suppression of RAS/ERK signaling, which NF1 loss overcame (Figs. 1f and 4b). NF1-GRD overexpression further decreased phosphorylated ERK and diminished Rb and cyclin A (Fig. 5c, d), indicating the NF1-GRD induces cell cycle arrest markers in p53-mutant AIS-bypassing cells. NF1-GRD overexpression increased SA-ßGal-positive and reduced EdU-positive cells (Fig. 5e–g). We also cultured 3D spheroids of FT282 cells coexpressing activated AKT and inducible NF1-GRD (Fig. S6a). Upon NF1-GRD induction, we observed fewer cells per spheroid and EdU-positive cells (Fig. S6b, c). Furthermore, NF1-GRD overexpression ablated anchorage-independent growth of the transformed p53-mutant FT282 myrAKT1-expressing cells (Fig. 5h). Collectively, these data highlight engagement of an intact NF1-dependent, p53-independent pathway reinstating AKT-driven senescence.

## Discussion

We surmised understanding AIS would uncover insights into potential tumor-suppressive and resistance mechanisms and provide new avenues for future therapeutic exploitation targeting PI3K/AKT/mTORC1-driven cancers.

RNA-seq and metabolomic analyses of cells undergoing AIS confirmed many hallmarks of prototypical OIS including cell cycle gene downregulation, SASP gene upregulation, lamin B1 downregulation, altered metabolism, and miR-146a upregulation upstream of NF-κB signaling [26]. However, our studies also identify specific mechanisms of AIS, consistent with our previous study showing AIS is p53-dependent and DNA damage independent [20]. We demonstrate AIS suppresses RAS/ERK signaling by upregulating inhibitors of RAS/ERK and NF1, distinct from the finding that constitutive BRAF signaling suppressed the PI3K/AKT/mTORC1 pathway to induce senescence [44]. Our finding that depleting NF1 caused AIS escape reinforces mutually exclusive senescence induction mechanisms. Restoring NF1’s ability to inhibit RAS/ERK signaling in NF1-depleted cells rescued AIS (Fig. 4e–i). Intriguingly, NF1 was posttranscriptionally upregulated during AIS but not OIS (Fig. 4a–c), which we hypothesize is due to enhanced synthesis or stabilization, as we showed for p53 [20].

Our findings also support the hypothesis that NF1-mediated suppression of RAS/ERK signaling is a key AIS tumor-suppressive mechanism. NF1 loss enhances transformation of p53-mutant fallopian tube epithelial cells expressing activated AKT (Fig. 5b). Conversely, overexpressing an NF1-GRD fragment reinstated senescence and blocked transformation (Fig. 5c–g). Consistent with this, a forward genetic screen in malignant peripheral nerve sheath tumors (MPNSTs) identified cooperation between *Pten* and *Nf1* loss, and reduced *PTEN* and *NF1* is associated with progression to high-grade MPNSTs [62].

As NF1 deficiency mediated escape from BRAF-driven OIS and BRAF inhibitor resistance in melanoma, which could be combatted with MEK inhibitors [18, 19], we hypothesize NF1 loss may be a key targetable mechanism of resistance to PI3K/AKT/mTORC1 inhibitors. While our findings and preclinical studies have demonstrated successful targeting of NF1 loss, clinical translation has proved challenging. However, the MEK inhibitor selumetinib has shown promise in clinical trials treating *NF1*-mutant neurofibromatosis and plexiform neurofibroma patients [63]. Inhibiting MEK could reinstate AIS in cancers with deregulated PI3K/AKT/mTORC1 signaling and NF1 loss. Our findings provide additional rationale for employing combination therapies targeting both the RAS/ERK and PI3K/AKT/mTORC1 pathways [64–66]. Indeed, a phase 2 clinical trial is planned combining selumetinib and the mTORC1 inhibitor sirolimus for MPNST patients [67].

We also functionally validated CCAR1 and FADD as novel AIS regulators. Potential therapeutic opportunities targeting them to reinstate AIS include a CCAR1 functional mimetic CFM-4 having in vivo efficacy in human nonsmall cell lung cancer treatment [68], and a dual-specific antibody against death receptors 4 and 5 upstream of FADD showing proapoptotic activity in human breast, ovarian, and colon cancer xenografts [69].

We identified substantial metabolic rewiring during AIS similar to that during OIS. Moreover, as 10% of the AIS escape screen hits are associated with metabolism, these data suggest metabolically reprogramming cancer cells with chronic PI3K/AKT/mTORC1 activation can re-engage AIS. This approach succeeded in a study of melanocytes expressing *BRAF*^*V600E*^, which undergo OIS and suppress the mitochondrial gatekeeper enzyme pyruvate dehydrogenase kinase 1 (PDK1) [70]. Inhibiting PDK1 caused *BRAF*^*V600E*^ inhibitor-resistant melanoma regression. From the TRACERx human lung cancer data analysis (Fig. 2e), we also found several AIS escape genes that could be involved in tumor evolution. Understanding how these genes regulate AIS will provide insight into the evolution of PI3K/AKT/mTORC1-driven cancers [2, 4, 7, 71].

**Fig. 6:**
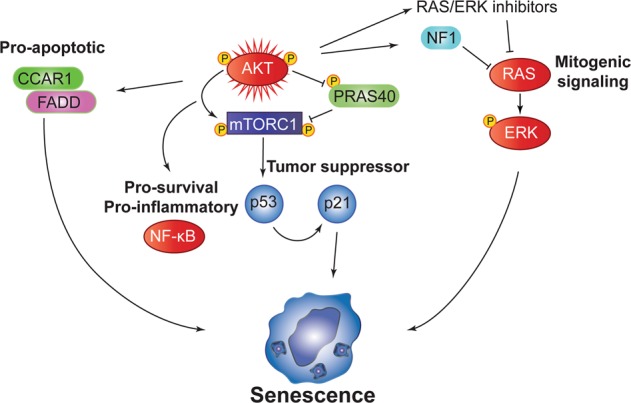
Proposed integrated model for regulation of AIS. Hyperactivation of AKT promotes enhanced mTORC1-dependent p53 synthesis, which transcriptionally upregulates p21 to induce proliferative arrest. Pro-inflammatory/survival NF-ĸB signaling is activated to drive the SASP as well as proapoptotic signaling through CCAR1 and FADD. Negative feedback suppression of RAS/ERK signaling maintains AIS. NF1 loss is sufficient to release negative feedback suppression of RAS/ERK signaling. Loss of these antiproliferative inhibitory signals leads to AIS escape, contributing to malignant transformation

A previous study demonstrated that while IR-induced senescent IMR-90 cells upregulated proapoptotic genes, FOXO4-dependent prosenescence signaling prevented apoptosis. Strikingly, FOXO4 inhibition promoted apoptosis [72]. Similarly, we hypothesize the AIS network harbors multiple signaling inputs including prosenescence and proapoptotic signaling (Fig. 6). Disrupting key pathways in the network can drive AIS escape, providing insight into tumor development and targeted therapy resistance mechanisms, which can potentially be harnessed to reinstate senescence or drive PI3K/AKT/mTORC1-driven cancers toward death.

## Supplementary information

Supplementary Figure Legends

Figure S1

Figure S2

Figure S3

Figure S4

Figure S5

Figure S6

Tables S1-S13
