# Supplementary material for: A functional genetic screen defines the AKT-induced senescence signaling network
Source: Cell Death Differ. 2019 Jul 8;27(2):725–41. doi: 10.1038/s41418-019-0384-8 (PMC7205866; doi:10.1038/s41418-019-0384-8)
Supplement: Supplementary file 1 — Supplementary Figure Legends [file 41418_2019_384_MOESM1_ESM.docx]

**Supplementary Figure Legends**

**Figure S1. miR-146a and SASP gene expression are upregulated during AIS.**

(a) Schematic of modified transcriptional reporter construct based on a miR-146a promoter-GFP fusion and a human phosphoglycerate kinase (PGK) promoter driving a constitutive Cherry fluorescent selectable marker. (b,c) BJ-TERT cells expressing the miR-146a promoter-GFP reporter were transduced with empty vector control or myrAKT1 and assessed by FACS analysis at 14 d post-transduction. (b) Representative FACS plot and (c) Quantification of GFP mean fluorescence intensity (MFI). Data are expressed as mean ± SEM. n = 3 experiments. *, *P <* 0.05 by two-tailed unpaired *t* test. (d) Heatmap of RNA-seq data showing common SASP gene expression and FC during AIS and OIS compared to proliferating cells. n = 3 biological replicates. (e-g) qRT-PCR showing relative mRNA expression normalized to *GAPDH* of BJ-TERT cells with empty vector pBabe, myrAKT1, or HRASV12 at 14 d post-transduction for (e) *CXCL1*, (f) *IL1A*, (g) *IL1B*. Data are expressed as mean ± SEM. n = 3 experiments. (*) *P* < 0.05 by one-way *ANOVA*.

**Figure S2. The metabolic profiles of AIS and OIS overlap.** (a) Principal component analysis of metabolite datasets from proliferating cells or those undergoing AIS or OIS. Note: The sample AIS-1 was excluded due to a technical issue in sample processing independent of sample quality. (b) Venn diagram showing common and distinct significant (BH adjusted < 0.05) metabolites. (c,d) Metabolite pathway enrichment comparing proliferating cells with (c) AIS or (d) OIS.

**Figure S3. RELA is required for AIS.** (a) Schematic of inducible mir30-based shRNA construct. The human phosphoglycerate kinase (PGK) promoter enables constitutive expression of enhanced blue fluorescent protein (EBFP2) and an internal ribosome entry site (IRES) allowing for translation of a reverse tetracycline transactivator (rTTA3). Doxycycline addition activates transcription at the TRE3G promoter to drive expression of red fluorescent protein (dsRed2) and the mir30-based shRNA. (b-i) BJ-TERT cells were transduced with either myrAKT1 or HRASV12 to promote AIS or OIS, respectively. Cells were seeded in colony formation assays and control (shREN) or RELA knockdown was induced with doxycycline. (b) Experimental design. (c) Western blots showing p65 expression in AIS or OIS cells. Actin was probed as a loading control. (d) Cells were stained for SA-ßGal activity or EdU. DAPI staining was used to visualize nuclei. Scale bars = 50 μm. (e,f) Quantification of percentage of cells with positive staining for (f) SA-ßGal activity or (g) EdU. (g) Crystal violet staining of colony formation assays. (h,i) Quantification of (h) colony area and (i) colony intensity). Data are expressed as mean ± SEM. n = 3 experiments. (**) *P* < 0.01; (***) *P* < 0.001 by one-way *ANOVA* as compared with corresponding shREN control. (j,k) BJ-TERT cells expressing inducible shRELA #2 were transduced with pBabe, HRASV12, or myrAKT1 without or with doxycycline induction of shRELA #2. (j) Cytokine membrane arrays from conditioned media of indicated cells. Common cytokines induced by either OIS or AIS are denoted in red. (k) Quantification of relative cytokine abundance normalized to cell number from AIS cells without or with shRELA #2 induction. Data are expressed as mean ± SEM. n = 3 biological replicates. (*) *P* < 0.05; (***) *P* < 0.001 by two-tailed unpaired *t* test.

**Figure S4. Validation of a subset of screen candidates in IMR-90 fibroblasts.** (a-d) Primary IMR-90 cells were transduced with empty vector control or myrAKT1. At 6 d post-transduction, cells were reverse transfected with 20 nM SMARTpool siRNAs and assessed 6 d post-transfection. (a) Western blots showing expression of the genes targeted by the siRNAs. Actin was probed as a loading control. (b) At 6 d post-transfection, cells were fixed and stained for SA-ßGal activity and EdU. Scale bars = 50 µm. (c,d) Quantification of percentage of cells with positive staining for (c) SA-ßGal activity or (d) EdU. Data are expressed as mean ± SEM. n = 3 experiments. (**) *P* < 0.01; (***) *P* < 0.001; (****) *P* < 0.0001 by one-way *ANOVA* as compared with the corresponding siOTP control.

**Figure S5. AIS escape signature in enriched in LUSC patients with CCAR1 or NF1 mutations.** Single sample gene set enrichment analysis (ssGSEA) of TCGA lung squamous cell carcinoma (LUSC) patients with *CCAR1* (n = 3) or *NF1* mutations (n = 2) as compared with patients without mutations. One-sided Wilcoxon tests were used to test for statistical significance.

**Figure S6. Overexpression of the NF1-GRD impairs the proliferation of myrAKT1-transformed FT282 spheroids cultured in Matrigel.** FT282 cells expressing NF1 shRNA #2, myrAKT1 and the inducible NF1-GRD fragment were cultured as 3D spheroids, embedded in Matrigel and treated in the absence or presence of doxycycline for 6 d. (a) Fixed spheroids were sectioned and stained for EdU. DAPI was used to stain cell nuclei. Scale bars = 50 μm. (b,c) Quantification of (b) the number of cells per spheroid and (c) percentage of EdU-positive cells. n = 16 (-Dox), n = 15 (+Dox). Data are presented as mean ± SD. (****) *P* < 0.0001, (**) P < 0.01 by two-tailed unpaired *t* test.
