# Supplementary figures and images for: A functional genetic screen defines the AKT-induced senescence signaling network
Source: Cell Death Differ. 2019 Jul 8;27(2):725–41. doi: 10.1038/s41418-019-0384-8 (PMC7205866; doi:10.1038/s41418-019-0384-8)

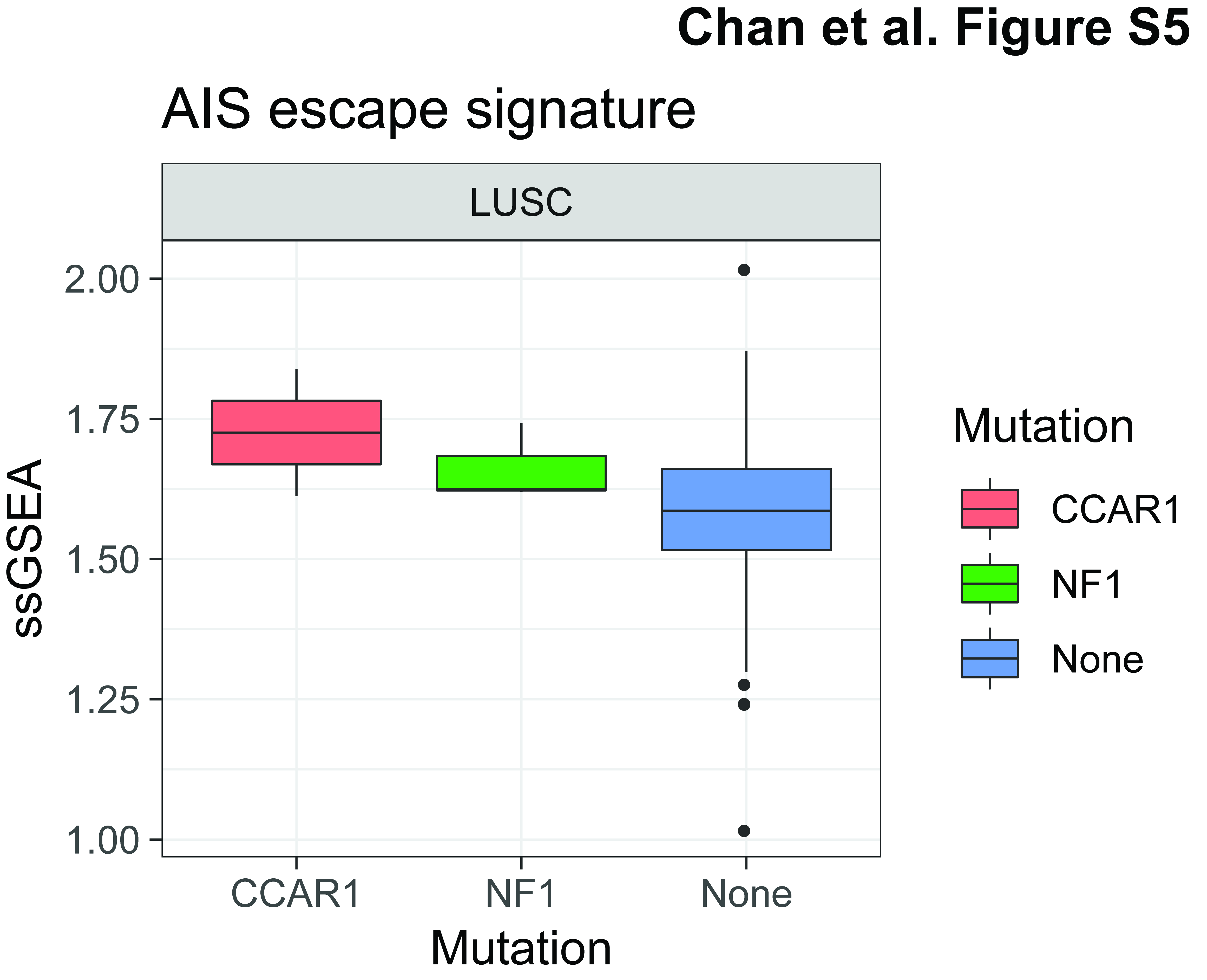

Supplement: Supplementary file 6 — Figure S5 [file 41418_2019_384_MOESM6_ESM.tif]

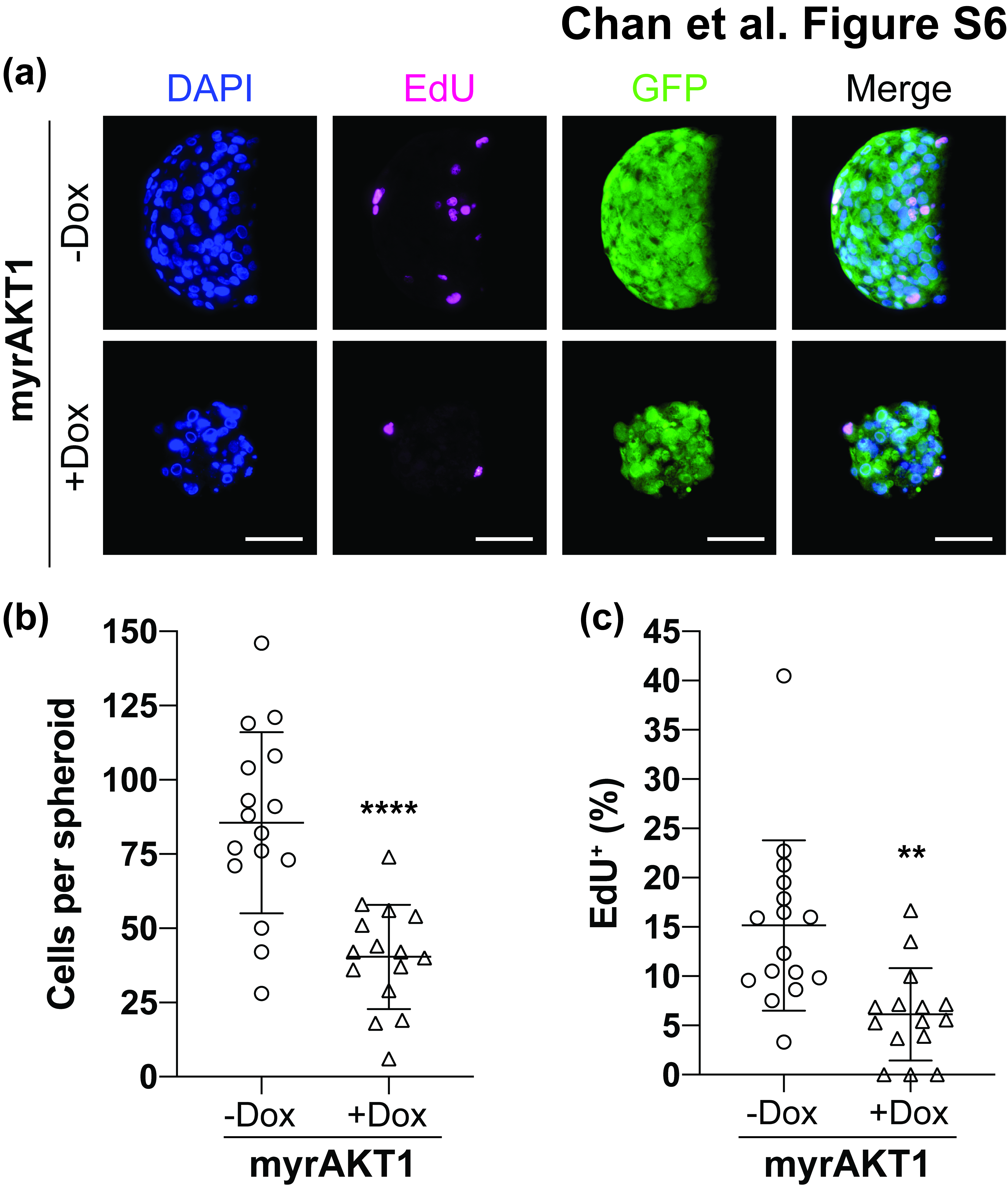

Supplement: Supplementary file 7 — Figure S6 [file 41418_2019_384_MOESM7_ESM.tif]
